# Supplementary material for: Content-rich biological network constructed by mining PubMed abstracts
Source: BMC Bioinformatics. 2004 Oct 8;5:147. doi: 10.1186/1471-2105-5-147 (PMC528731; doi:10.1186/1471-2105-5-147)
Supplement: Additional File 2 — The original results of the above study (non-essential files are deleted to keep the file size under the limit set by BMC bioinformatics). [file 1471-2105-5-147-S2.bz2 › chilibotAdditionalFile2/dip30/514ID8799122E10817/html/P300_ESR1.html]

 


 **P300** and **ESR1** 
  
Found 50 abstracts in PubMed, retrieved 30.  
 

 What does Google say? 
 PDF only 
| .edu only 

---

**Interactive relationship** (e.g. stimulation, inhibition, etc)

**Inhibitory relationship**- **p300**  Modulates the BRCA1 inhibition of estrogen receptor  [ **ESR1** ]  activity.  Ref: 11782371 Cancer Res, 2002
- These findings suggest that the cofactors  **p300**  and CBP modulate the ability of the BRCA1 protein to inhibit ER alpha  [ **ESR1** ]  signaling.  Ref: 11782371 Cancer Res, 2002
- They further suggest that the BRCA1 inhibition of ER alpha  [ **ESR1** ]  activity may be attributable, at least in part, to the down regulation of  **p300** .  Ref: 11782371 Cancer Res, 2002
**Neutral relationship**- pRb2 p130 E2F4 5 HDAC1 SUV39H1  **p300**  and pRb2 p130 E2F4 5 HDAC1 SUV39H1 DNMT1 multimolecular complexes mediate the transcription of estrogen receptor alpha  [ **ESR1** ]  in breast cancer.  Ref: 12789259 Oncogene, 2003
- Mediator and  **p300**  CBP steroid receptor coactivator complexes have distinct roles, but function synergistically, during estrogen receptor alpha  [ **ESR1** ]  dependent transcription with chromatin templates.  Ref: 12482985 Mol Cell Biol, 2003
- We now report that the ability of BRCA1 to repress ER alpha  [ **ESR1** ]  activity correlates with its ability to induce down regulation of the cellular levels of the transcriptional coactivator  **p300**  in breast and prostate, but not in cervical cancer cells.  Ref: 11782371 Cancer Res, 2002
- The rescue function mapped to the cysteine histidine rich domain CH3, a region of  **p300**  CBP that we found to interact directly with the conserved COOH terminal activation domain AF 2 of ER alpha  [ **ESR1** ] .  Ref: 11782371 Cancer Res, 2002

**Non-interactive relationship** (e.g. studied together, co-existance, homology, etc.)

- We show that in contrast to SRC1, direct binding of CBP to the estrogen receptor  [ **ESR1** ]  is weak, suggesting that SRC1 functions primarily as an adaptor to recruit CBP and  **p300** .  Ref: 11113179 Mol Cell Biol, 2001
- We have used a biochemical approach, including an in vitro chromatin assembly and transcription system, to examine the functional role for Mediator in the transcriptional activity of estrogen receptor alpha  [ **ESR1** ]  ERalpha with chromatin templates, as well as functional interplay between Mediator and  **p300**  CBP during ERalpha dependent transcription.  Ref: 12482985 Mol Cell Biol, 2003
- We investigated in vivo occupancy of ER alpha  [ **ESR1** ]  promoter by pRb2 p130 E2F4 5 HDAC1 SUV39 H1  **p300**  and pRb2 p130 E2F4 5 HDAC1 SUV39H1 DNMT1 complexes, and provided a link between pRb2 p130 and chromatin modifying enzymes in the regulation of ER alpha  [ **ESR1** ]  transcription in a physiological setting.  Ref: 12789259 Oncogene, 2003
- We also found that the association of the co regulatory protein CBP  **p300**  with ER alpha  [ **ESR1** ]  and NF kappa B was increased by spermine treatment of MCF 7 cells.  Ref: 11313919 Oncogene, 2001
- ... two other nuclear receptor coactivators, the  **p300**  CBP associated factor PCAF and the glucocorticoid receptor interacting protein 1 GRIP1, failed to rescue the ER alpha  [ **ESR1** ]  activity.  Ref: 11782371 Cancer Res, 2002
- In vivo and in vitro binding assays revealed that while 4 hydroxy tamoxifen OHT induced binding of ER alpha  [ **ESR1** ]  to both an AF 1 coactivator complex p68 p72 and  **p300**  and corepressor complexes N CoR SMRT, BBP selectively enhanced the binding to the AF 1 coactivators.  Ref: 12738788 J Biol Chem< MedlineTA>J Biol Chem, 2002
- Synergistic, p160 coactivator dependent enhancement of estrogen receptor  [ **ESR1** ]  function by CARM1 and  **p300** .  Ref: 11010967 J Biol Chem, 2000
- **p300**  and ER alpha  [ **ESR1** ]  were also found to interact in vivo and to colocalize within the nucleus in breast cancer cells.  Ref: 11782371 Cancer Res, 2002
- Further experiments demonstrated that overexpression of the transcriptional coactivator cAMP response element binding protein CREB binding protein CBP  **p300** , a factor common to both the estrogen receptor  [ **ESR1** ]  and HIF pathways, eliminated E2beta mediated attenuation of hypoxia induced ET 1 promoter activity.  Ref: 12060564 Am J Physiol Lung Cell Mol Physiol, 2002
- The current study explored the cooperative functional and mechanistic relationships among GRIP1, CARM1, and  **p300**  in transient transfection assays, where they enhanced the ability of the estrogen receptor  [ **ESR1** ]  ER to activate transcription of a reporter gene.  Ref: 11010967 J Biol Chem, 2000
- In addition, polyamine analogs inhibited the association of ER alpha  [ **ESR1** ]  and NF kappa B with CBP  **p300**  and were unable to facilitate nuclear translocation of NF kappa B.  Ref: 11313919 Oncogene, 2001
- Histone H1 exerts its repressive effect without inhibiting the sequence specific binding of ER alpha  [ **ESR1** ]  to chromatin or the overall extent of targeted acetylation of nucleosomal histones by the coactivator  **p300** .  Ref: 11909941 Mol Cell Biol, 2002
- Direct acetylation of the estrogen receptor alpha  [ **ESR1** ]  hinge region by  **p300**  regulates transactivation and hormone sensitivity.  Ref: 11279135 J Biol Chem, 2001
